# Sudden gamer death: non-violent death cases linked to playing video games

**DOI:** 10.1186/s12888-022-04373-5

**Published:** 2022-12-23

**Authors:** Diana Kuperczko, Peter Kenyeres, Gergely Darnai, Norbert Kovacs, Jozsef Janszky

**Affiliations:** 1 Department of Neurology, Medical School, University of Pecs, Pecs, Hungary; 2 1st Department of Medicine, Medical School, University of Pecs, Pecs, Hungary; 3 Department of Behavioural Sciences, Medical School, University of Pecs, Pecs, Hungary

**Keywords:** Internet gaming disorder, Video game, Death, Acute autonomic dysfunction

## Abstract

**Background and aims:**

Internet gaming disorder (IGD) is an emerging problem. Rarely, media reports about people, who have died during playing video games, but thus far no systematic, scientific study is available about the topic. We investigated such cases, looking for common characteristics, connection between gaming and death, and the possible reasons leading to death.

**Methods:**

Cases were collected through internet search with general keywords, with ones specific to identified cases, and by working along cross references.

**Results:**

24 cases were found: one from 1982, the others between 2002 and 2021. Twenty-three of the victims were male, age ranged from 11 to 40 years. More than half of the cases originated from Southeast Asia, and 12 deaths happened in internet cafes. Gamers played action-rich multiplayer games. In 18 cases the gaming session before death was extremely long (around a day or even several days) with minimal rest. The cause of death was pulmonary embolism in 5 cases, cerebral hemorrhage in 2 cases, most of the rest was presumably due to fatal cardiac arrhythmia.

**Discussion:**

Long sedentary position and dehydration may precipitate thromboembolism, acute blood pressure elevation during gaming may promote cerebral hemorrhage, and several factors (including acute and chronic sleep deprivation, exhaustion, stress) can lead to acute autonomic dysfunction and fatal arrhythmia.

**Conclusion:**

Incidence of non-violent death cases linked to playing video games is presumably very low. It mostly occurs in young males and it is often characterized by extremely long gaming time.

**Supplementary Information:**

The online version contains supplementary material available at 10.1186/s12888-022-04373-5.

## Background

Today video games provide relaxation and entertainment for billions with challenges to fulfill, new experiences to meet and new frontiers to explore. But attraction sometimes escalates to obsession or addiction. In the past two decades, year by year, reports could be read about people dying during playing video games, mostly young ones who had been playing for an extremely long period. Other reports cited homicide or suicide cases, where video games were involved in the motivation. The grim suggestion “video games may suck you in and cost your life” could polarize public opinion leading to exaggeration and overreaction.

Between 1995 and 2017 penetration of internet use raised from below 1–46% of the world population, being much higher in developed countries among younger age groups [1]. Growing of internet use was accompanied by growing of internet addiction. Addicted one can be overusing several modalities of internet (generalized internet addiction) or be focused on a specific internet-based activity like playing online video games. The latter phenomenon was nominated Internet gaming disorder (IGD) in the Diagnostic and Statistical Manual of Mental Disorders (DSM-5) in 2013 [2]. A metaanalysis of 113 epidemiological studies between 1996 and 2018 from 31 nations reported the averaged prevalence of generalized internet addiction to be 7% in the population, while the individual sources reported it wildly variable between 0.5 and 40% due to different definition and measurement method. An increasing tendency was demonstrated through years. IGD prevalence (based on sources from 2015 to 2018) was 2.5% (0.2–14.9%) [3]. The prevalence depends on the availability of internet and the internet culture, being much higher in Southeast Asia, where the prevalence of IGD among adolescents was estimated to be 5% according to a paper from 2014, and the prevalence is seven times higher among male adolescents than female adolescents [4]. Someone with IGD often spend several hours a day gaming, but in extreme cases the gamer may spend days online, consecutively, with little food or drink intake or sleep, which may lead to adverse (or fatal) physiological conditions [5].

## Aims

While extensive debates can be found on the internet about the topic, according to our knowledge, it has not been systematically reviewed in the medical or health science literature, nor have we found any studies or case reports. We aimed to investigate the real size of the problem of death linked to playing video games, if these subjects were addicted to gaming as often stigmatized, the connection between gaming and death, and the possible reasons leading to death.

## Methods

We tried to collect all cases, where video gaming was associated with the death of the gamer. Search was started on the internet browsing (Google) for keywords “death”, “die” and “video game”. We found several cases and case compilations in online newspapers, blogs, and video portals. Obtaining specific keywords for individual cases, we gathered as much information as possible about them, preferring trustable online newspaper articles. Considerable cross reference was found among sources, which we could work along. We ended up with a moderately low number of detailed cases, which were frequently mentioned in several online newspaper and cited in compilations, and a few brief, undetailed references, which could not be further elaborated. We did not consider other death cases that could be associated to video games (accidents, suicides or homicides fueled by impulse, rage, revenge, frustration, negligence, carelessness or a game motivated pattern), only where the player itself died presumably connected to the action of gaming. Scientific search (Pubmed) yielded no relevant publications.

## Results and discussion

Using the mentioned method, we found 24 relevant cases up until the end of 2021, and numbered them in chronologic order. We did not anonymize the victims since their names are publicly available. Cases are summarized in Table 1, highlighting specific characteristics, discussed later. More detailed descriptions of the cases are available in the Supplementary material 1. An isolated case was reported in the early 1980’s, but the cases of real interest started after 2002, with 1 or 2 cases every year or two years, but without trend for remarkably increasing incidence.

**Table 1:** Summary of cases

**Case number**	**Case No. 0**	**Case No. 1**	**Case No. 2**	**Case No. 3**	**Case No. 4**	**Case No. 5**	**Case No. 6**	**Case No. 7**	**Case No. 8**
**Name**	Jeff Dailey	Peter Burkowski	Kim Kyung-Jae	Seung Seop Lee	Zhang	“Chinese man”	Tim Eves	“Chinese man”	Chris Staniforth
**Date of death**	1981 January	1982.04.03	2002 October	2005.08.05	2007.02.24	2007.09.15	2009.03.04	2011 February	2011 May
**Country**	USA (Chicago)	USA (Chicago)	South Korea (Gwangju)	South Korea (Taegu)	Northern China (Jinzhou)	Southern China (Guangzhou)	UK (Hopton-on-Sea)	China (outskirts of Beijing)	UK (Sheffield)
**Age**	19	18	24	28	26	30	25	30	20
**Gender**	male	male	male	male	male	male	male	male	male
**Game played**	Berzerk	Berzerk	Mu	StarCraft	World of Warcraft	unknown	Wii Fit	unknown	Halo
**Was it consecutive?**	no	no	yes	yes	yes	yes	no	yes	yes
**Length of gaming**	unknown	less than 30 min	86 h	50 h	7 days	3 days	few hours?	3 days	often 12 h
**Did he/she stand up during gaming?**	playing standing	playing standing	paused only to purchase cigarettes and to use the bathroom	took only restroom breaks, ate and drank very little, had brief naps	stopping only for eating, toilet breaks, and a few hours of sleep	without considerable breaks	actively moving during the game	without eating and sleeping	unknown
**Did he/she miss sleep?**	no	no	yes	yes (2 days)	yes, had only few hours of sleeps	yes, minimal breaks	unknown	unknown	unknown
**Was he/she gaming when he/she died?**	unknown	after gaming	yes	yes	yes	yes	yes	yes	no
**Did he/she eat/drink?**	unknown	unknown	yes	very little	yes	unknown	unknown	unknown	unknown
**On what platform did he/she play?**	arcade machine	arcade machine	PC	PC	PC	PC	Wii Fit games console	unknown	Xbox
**Circumstances of death**	unknown	He set up two top-ten scores in the game, then turned to another arcade machine, collapsed	unknown	He fell off his chair onto the floor; conscious with closed eyes. Died in hospital hours later	Parents witnessed him twitching and slumping over his computer screen. Resuscitation attempt was unsuccessful.	He fainted. Paramedics tried to revive him but failed.	He just returned from Portuguese a few hours before. Suddenly collapsed and died while jogging on the Wii Fit game.	lost consciousness	He collapsed after an interview at a JobCentre. Previously he was woken in the night by a ‘strange feeling’ in his chest
**Suspected cause of death**	unknown	heart attack	pulmonary embolism	heart failure	overwork and obesity	exhaustion	Sudden Arrhythmia Death Syndrome	unknown	deep vein thrombosis, pulmonary embolism
**Location of death**	arcade	arcade	internet café	internet café	at home	internet café	at home	internet café	JobCentre
**Was he/she a game addict?**	no	no	yes	yes	yes	unknown	unknown	unknown	yes
**Other risk factors**	unknown (some source report obesity)	no alcohol or drugs	smoking	unknown	extreme obesity (ca. 150 kg)	unknown	unknown	unknown	seems obese on his photo
**History of diseases**	unknown	autopsy found scar tissue on his heart, older than 2 weeks	unknown	wearing glasses	unknown	unknown	unknown	unknown	no
**Case number**	**Case No. 9**	**Case No. 10**	**Case No. 11**	**Case No. 12**	**Case No. 13**	**Case No. 14**	**Case No. 15**	**Case No. 16**	**Case No. 17**
**Name**	Anna-Lee Kehoe	Chen Rong-Yu	Chuang	Chang	Wang	Chu	Hsieh	Wu Tai	Rustam
**Date of death**	2011.07.22	2012.02.01	2012.07.15	2013 August	2014 February	2015.01.01	2015.01.08	2015 March	2015.09.01
**Country**	UK (Bridport)	Taiwan (New Taipei City)	Taiwan (Tainan)	Taiwan (Hualien City)	Taiwan (Greater Kaohsiung)	Taiwan (New Taipei)	Taiwan (Kaoshiung)	China (Shanghai)	Russia’s Republic of Bashkortostan
**Age**	13	23	18	35	40	38	32	24	17
**Gender**	female	male	male	male	male	male	male	male	male
**Game played**	unknown	League of Legends	Diablo III	unknown	unknown	unknown	combat games	World of Warcraft	Defence of the Ancients
**Was it consecutive?**	unknown	yes	yes	yes	yes	yes	yes	yes	yes
**Length of gaming**	unknown	23 h	40 h	10 h	13 h	5 days	3 days	19 h	22 days
**Did he/she stand up during gaming?**	yes	unknown	unknown	unknown	unknown	yes	unknown	no	yes
**Did he/she miss sleep?**	unknown	little sleep in front of the monitor	yes	unknown	unknown	yes	yes, taking only naps sitting in the chair or laying on the desk	unknown	unknown
**Was he/she gaming when he/she died?**	yes	yes	no	probably	yes	no	yes	yes	yes
**Did he/she eat/drink?**	unknown	unknown	no	unknown	unknown	yes	unknown	no	yes
**On what platform did he/she play?**	Xbox	PC	PC	unknown	unknown	unknown	unknown	PC	PC
**Circumstances of death**	She stood up from gaming to say ‘Mum, I can’t breathe’, then collapsed. She was resuscitated but remained brain-dead	An employee tried to wake him, but found him dead, still reaching for the keyboard	He was found resting on the table. After the staff woke him up, he took a few steps and collapsed, then died shortly after arrival to hospital.	unknown	Attendants found Wang dead, but his eyes were still open and staring at the screen as though he were still playing	found dead in the establishment’s bathroom	Security camera footage showed Hsieh struggled with chest pains before collapsing	He started to cough violently, coughed up blood then slumped back.	unknown
**Suspected cause of death**	heart attack caused by asthma	cardiac arrest	thromboembolism	unknown	unknown	his medical condition, physical exhaustion	cardiac arrest	probably pulmonary embolism	thromboembolism due to broken leg
**Location of death**	at home	internet café	private room of an internet café	internet café	internet café	internet café	internet café	internet café	at home
**Was he/she a game addict?**	unknown	unknown	unknown	unknown	unknown	unknown	yes	yes	yes
**Other risk factors**	no	unknown	unknown	unknown	unknown	smoking	unknown	unknown	broken leg
**History of diseases**	asthma	treated for a heart attack last year	unknown	unknown	unknown	taking medicines for liver disease, gallstones	unknown	unknown	unknown
**Case number**	**Case No. 18**	**Case No. 19**	**Case No. 20**	**Case No. 21**	**Case No. 22**	**Case No. 23**	**Case No. 24**		
**Name**	Brian Vigneault	Bogdan Akh	Fahad Fayyaz	Natsupon Arunrat	Piyawat Harikun	Muhammad	Dharshan		
**Date of death**	2017.02.19	2018.10.29	2019.02.05	2019.04.27	2019.11.04	2020 October	2021.02.01		
**Country**	USA (Virginia)	USA (San Jose) (but gamer was Swedish)	Pakistan (Lahor)	Thailand (Samut Prakan)	Thailand (Udon Thani)	Egypt	India (Puducherry)		
**Age**	35	21	11	32	17	12	16		
**Gender**	male	male	male	male	male	male	male		
**Game played**	World of Tanks	Fortnite	Fortnite	unknown	combat games	PlayerUnknown’s Battlegrounds	Fire Wall		
**Was it consecutive?**	yes	no	yes	yes	yes	unknown	unknown		
**Length of gaming**	22 h	several hours a day in the past 2 months	a few hours	probably 12 h	all-night gaming sessions continued through the day	unknown	4 h		
**Did he/she stand up during gaming?**	yes	unknown	unknown	unknown	yes	unknown	unknown		
**Did he/she miss sleep?**	unknown	unknown	probably not	unknown	yes	unknown	probably not		
**Was he/she gaming when he/she died?**	no	no	yes	yes	yes	yes	yes		
**Did he/she eat/drink?**	unknown	yes	unknown	unknown	yes (There was a pile of takeaway boxes on his desk.)	unknown	unknown		
**On what platform did he/she play?**	PC	PC	PC/Xbox?	PC	PC	mobile phone	mobile phone		
**Circumstances of death**	He was doing a charity 24-hour-streaming. He left for a cigarette break but never returned.	Died during sleeping after a gaming competition after 2 months of intensive training	Parents later found him unconscious in his room with a controller in his hand.	He was found dead probably 4 h after having died, laying back on the chair, his mouth covered with blood from biting his tongue.	Father found his son collapsed from the computer chair and was slumped against a PC tower on the floor.	Parents found him unresponsive with his mobile phone open to the game. He was dead before arriving to the hospital.	He collapsed while playing the game. He was taken to hospital, but doctors did not succeed in reviving him.		
**Suspected cause of death**	confirmed fentanyl overdosing	unknown	heart attack	cardiac arrest	stroke	cardiac arrest	cerebral haemorrhage		
**Location of death**	at home	at hotel	at home	internet café	at home	at home	at home		
**Was he/she a game addict?**	gaming professional	unknown	yes	yes	yes	yes	unknown		
**Other risk factors**	heavy smoker	unknown	unknown	unknown	unknown	obesity	unknown		
**History of diseases**	unknown	unknown	unknown	heart disease and high blood pressure	unknown	unknown	unknown		

### Demographic and geographic characteristics

Only one of the reported victims was female (No. 8), but we do not know about the length and intensity of her gaming.

Internet addiction among females usually involves addiction to social media rather than gaming - IGD is seven times higher among male than female adolescents [4]. It seems that deaths were connected to action-rich games (see below), which are less popular among females. Competitiveness in games is more common among males, which may bind them for a long period to the game without pausing.

The victims were mostly adolescents or young adults (age ranging from 11 to 40 years). The first few victims died in their twenties; this was the age group that primarily got acquainted with computer games, and in whom IGD became an emerging problem. In the following years, age of victims broadened. The former teens and twenties aged to thirties. Further spread of computers, internet and games reached the very young age group, as well – nowadays kids often start to play video games before learning to read, and get their game-able smartphone before 10 [6].

More than half of the cases originate from Southeast Asia (Taiwan, China, South Korea, Thailand) and most of them (12 cases) happened in internet cafes. It is not that surprising according to the popularity of these places [7]. Buying gaming hours are cheap, and are far more economical alternative for young players than paying for an own PC, broadband internet connection, electricity and the games themselves [8]. Moreover, internet cafes provide cheap food and drinks, some even private rooms with beds to sleep or to shower. With 0–24 opening hours a customer may not even need to move out for days. Without supervision, young people can get away from their parents, escape reality, and get lost in the game undisturbed without thinking about the pressures of school and work [8]. On the other hand, in the cases from the western region the gaming event happened at home, as internet cafés never had such high popularity, due to the expansion of home-based e-mail and internet access points [7]. In the recent years rapid spread of smartphones has been restructuring Internet access and gaming, as well, and the first gaming deaths linked to smartphones also appeared.

### Incidence of death by gaming

Using the above mentioned data, we tried to give a very rough estimation about the incidence of gaming related death. Number of internet users is growing, being around 2 billion (28% of 7 billion people in 2010) and 3 billion (41% of 7.38 billion people in 2015) ([9, 10]). Risk population seems to be males from 15 to 40 years, so about 360–540 million; we used 450 million for further calculation.

Using the 2014 Asian data, the prevalence of IGD among adolescents was estimated to be 5%, but since most of them were males, we used 10% for males. The population at risk (adolescent males with IGD) may be around 45 million. This means a roughly 1 death per 2 million gamers over about 15 years course, or 1 per 30 million yearly. Considering only the most avid gamers the incidence is probably higher.

### Games involved in death cases

In most cases the name (or the category) of the game the victim had been playing was reported. Detailed description of these categories is provided in the Supplementary material 1. The common feature of these games is that they all need strong, strenuous mental concentration. The gamer may lock out the disturbing outside world, lose track of the passing time, and dimly notice hunger, thirst, tiredness or discomfort, which may lead to the adverse consequences. No one was reported to die while playing a *sandbox* game (like The Sims, Minecraft) [11], where player use creativity instead of action, or an *adventure* game [12], which involves exploring and playing along a storyline (sprinkled with action and dexterity elements) in an enjoyably slow pace instead of the aforementioned rush. The games played were definitely or presumably multiplayer games, where the competitive factor or adaptation to the other players enforces the gamer into a faster pace and prevents him from stopping, contrary to single-player mode, where one can do it in his own pace.

### Cause of death

Ten victims were reported to have co-morbidities or risk factors: extreme obesity (No. 4), obesity (No. 8 and 23), asthma (No. 9), previous heart attack (No. 10) or heart problem (No. 21), high blood pressure (No. 21), liver disease/gallstones (No. 14), broken leg (No. 17), smoking (No. 2, 14 and 18), in the other cases such risk factors were not known.

Five sudden deaths were caused by pulmonary embolism (PE). A critically large embolus can lead to hemodynamic collapse, shock and death, which happens in about 10% of the cases [13]. Autopsy can unequivocally prove or rule out the embolism, but preceding symptoms (sudden onset, stabbing chest pain, dyspnea, coughing up blood, swollen leg) may suggest the diagnosis, as well. Prolonged immobilization, bedrest or long sitting and dehydration are well-known risk factors for deep venous thrombosis (DVT) in the lower limb. “Economy class syndrome” refers to DVT of aircraft passengers, where prolonged sitting cause venous stasis, may damage the endothelium of the veins, while dehydration due to dry atmosphere or drinking less, further increase risk of thrombus formation. Aforementioned gamers were similarly sitting long and moving little. Focused on the game they may be less aware of being stiff and sore, or thirsty. Obesity and smoking are further relevant risk factors. After thrombus formation, PE may strike later or in waves, like in case of Chris Staniforth (No. 8), who mentioned ‘strange feeling’ in his chest the previous night, and died at the JobCentre next day. Besides 22 days of sedentary gaming, Rustam (No. 17) had a broken leg, which increases risk of DVT over 10-fold on its own [14]. Besides the extent of embolism, survival is determined by the ability to compensate for the increased resistance of pulmonary circulation, requiring good right ventricular function with adequate preload and autonomic regulation. Young subjects are expected to have better chances than elder ones. Autonomic dysfunction (explained later) and hypovolemia due to dehydration may have contributed to the death of the gamers.

In one case (No. 22) stroke in another (No. 24) cerebral haemorrhage was reported as cause of death. Stroke was not further specified. It might have been ischemic stroke, which often causes disability but rarely sudden death, and is very hard to be explained at this age. It more probably could have been haemorrhagic stroke or subarachnoid haemorrhage. Haemorrhagic stroke in young adults can be caused by hypertension, vascular malformation, drug abuse, venous sinus thrombosis, hematologic conditions. According to the bleeding location and size, it may lead to herniation, coma or death. Subarachnoid haemorrhage is due to the rupture of saccular aneurysms in more than half of all cases. 25% of these patients die within 24 h of the event; many die before they reach the hospital. Hypertension or acute elevation in blood pressure, smoking are risk factors for aneurysmal haemorrhage [15].

In many cases (No. 1,3,6,9,10,15,20,21,23) ‘heart failure’, ‘heart attack’ or ‘cardiac arrest’ was named as cause of death, though the terms may not have been medically properly used. In other cases, definite cause of death was not even specified or speculations (like “overwork and obesity”, “fatigue”) were reported. Sudden cardiac death (SCD) developing such rapidly almost always happen due to onset of malignant ventricular arrhythmia (ventricular fibrillation, extremely fast ventricular tachycardia) or sudden severe dysfunction of the sinus or AV node without escape rhythm. Other possible specific causes (like extreme extent myocardial infarction and loss of pump function or aortic dissection leading to rapid bleeding or pericardiac tamponade) are improbable and could be easily identified during autopsy. Arrhythmia on the other hand often lacks obvious structural background. However, autopsy results or background medical information were available only in few cases, mostly the cases from the Western countries. For the Asian cases these were either not performed, or the international news agencies were not interested in writing follow-up articles. In many cases excitement, fatigue, physical exhaustion, sleep deprivation, dehydration, lack of movement, were named as contributing factors to the cardiac death.

Some cases seem out of the pattern. Tim Eves (No. 6) was playing on Wii Fit, where player actually moves and jogs, turning gaming into a sport, a mild to vigorous aerobic training. Circumstances did not suggest anything extraordinary either. His death is rather sport-related than game-related. Anna-Lee Kehoe’s (No. 9) symptoms did not resemble an asthma attack, even complete airway obstruction would result in a longer agony. Symptoms rather suggest massive pulmonary embolism, and the source did not mention if examinations or autopsy was performed to rule it out. On the other hand, thrombosis at such young age is unlikely without the presence of thrombophilia. Though no obvious provoking factors were mentioned, arrhythmia-related death (heart attack) is possible in the presence of underlying disease (like arrhythmogenic right ventricular cardiomyopathy or channelopathy). Similarly, in Burkowski’s case (No. 1) the half hour gaming session must have been no overexertion or overly stressful for the experienced player (who set up two top-10-scores). The scar on his heart, that probably was involved the lethal arrhythmia, is of unknown origin: cardiomyopathy, peri/myocarditis, mechanical injury or an unlikely myocardial infarction? He probably could have died due to any other physiological stressors or excitement, as well. Similarly, in other cases (No. 20, 21, 24), though the victims were gaming addicts, no extremity was reported about the last gaming session. If there is a very low chance of death during a gaming session, there is obviously a higher cumulative chance for ones who repeatedly do it.

Bogdan Akh’s (No. 19.) death is not connected to uncontrolled gaming binge, but to a scheduled training for a competition, and we may consider it the first eSport related death (see below). Extreme exhaustion is unlikely, it was probably avoided in favour of the best performance on the competition. Strangely, death occurred not around the training or the competition, but after them, during rest, when all stress subsided. Could this change in balance be the trigger, or were there other possible factors, like a celebration after the competition?

### Sleep deprivation and death

In 18 cases the gaming session before death was extremely long (around a day or even several days) with minimal rest, which results in acute sleep deprivation, or the victim was repeatedly playing quite long sessions, which suggests chronic sleep deficiency.

Sleep is a natural relaxing state for regeneration [16]. Uncomfortable effects of acute or chronic sleep deprivation (SD) are experienced by almost everyone; impaired concentration, reduced mental and physical performance, mood alterations, irritability, to mention some. They may indirectly lead to fatal consequence, like falling asleep and causing a traffic accident. It is another question though if or when can SD itself trigger or contribute to a biological shock resulting in death, like in the aforementioned gamers. In an animal model of acute SD, all rats died within 2–6 weeks of continuous or paradoxical SD [17]. The animal lost weight despite increased food intake, showed altered thermoregulation, but no obvious changes in brain morphology or function could be demonstrated.

Experimental data of such drastic SD in human is not available. In a study, subjects after a night with sleep dept (still 1.7 h sleep on average) showed increase of the QT interval and QT dispersion (still within the normal range), which may make more susceptible to lethal ventricular arrhythmias [18]. A case report demonstrated spontaneous coronary dissection and myocardial infarction after 72 h SD due to overtime work [19].

Several longitudinal human studies demonstrated a U-shaped association between sleep duration and all-cause mortality, optimal amount being around 7 h sleep per day, with increased mortality of both short and long sleepers [20, 21, 22, 23, 24]. Besides direct effect of SD, short sleep may also contribute to development of physiological and social outcomes that may lead to increased mortality; e.g. cardiovascular disease, stroke, dyslipidemia, diabetes, hypertension, obesity, cancer, presence or development of stress, altered inflammatory cytokine levels and impaired immune response [25, 26, 27]. Exact sleep habits of the gamers are not known, but it is presumable that the ones who were indicated as gaming addicts, and were doing regular several-day-long gaming binges or over 10-hour daily gaming sessions, were also taking away time from sleeping in favour of gaming, and are on the short-sleeper arm of the curve. The above results are also based on mainly middle-aged or elder subjects, and applying them on the mostly young gamers is questionable. On the other hand, chronic short-sleeping may have already done subclinical damage in these individuals, making them more susceptible to an acute event.

### Sport and sudden cardiac death

Eventual, unexpected, sudden cardiac death (SCD) cases are reported in another field, as well: sports and athletes. We tried to apply such experiences to our population. Public and medical attention is high for such events, the cases and causes are usually thoroughly investigated. Widespread registers offer more reliable data in contrast to the cases of video gamers. Due to inconsistencies of definitions incidence widely vary between 1 in 3000 and 1 in million athlete per year [28]. Higher incidence was found among males (3–5 times) and blacks (3 times) [29]. Deaths occur most commonly in team sports, which have the highest levels of participation [30]. In a study, media database reports identified 70% of the total SCD cases, suggesting the high media attention of such unexpected death on young ones [29]. In many cases structural abnormalities (hypertrophic cardiomyopathy or idiopathic left ventricular hypertrophy, dilated and restrictive cardiomyopathy, arrhythmogenic right ventricular cardiomyopathy, congenital coronary anomalies, aortic rupture due to Marfan syndrome, myocarditis, valvular disease, commotio cordis) were present, but individuals with morphologically normal hearts were common, as well, referred to as sudden arrhythmic death syndrome (SADS). In some of them primary electrical disorders or channelopathies (like Brugada syndrome, Wolff–Parkinson–White syndrome, long QT syndromes) could be identified, but about 30% remained unexplained.

### eSports

Outstanding performance attracts audience in gaming, as well, which up today turned into a rapidly growing industry, eSport, with over 1 billion audience [31], and with gamers doing it as a full-time profession. Average eSport player practices around 5.5 h a day and even 10 h a day prior to competitions. 15% reported 3 h or more of sitting and playing without standing to take a break. This sitting for hours staring the monitor with several hundred clicks and keypresses per minute results in chronic overuse (and sometimes career-ending) injuries (eye fatigue, neck or back pain, wrist and hand pain), but one case of deep venous thrombosis was also reported [31]. As far as we know, up until today no case of death among eSport athletes linked to gaming was officially reported. On the other hand, eSport community is far smaller, and exists for a shorter time than the general gamer and IGD population, and the lower numbers may have just not produced a death case.

### Stress and cardiac death

Death of Chen Rong-Yu (No. 10), was a well investigated, still unexplained case, about which we have more detailed information from an interview with his physician, Dr. Ta-Chen Su in the book Death by video game [32]. Rong-Yu had had a heart attack 3 months before. Extensive examinations (including ECG, echocardiography, 24-hour ECG, coronary angiography, cardiac electrophysiology study) did not show underlying abnormalities. Cardioverter defibrillator implantation was suggested which Rong-Yu refused. Dr. Su provided further hypotheses about his death, elaborated below.

Most organs are regulated by the sympathetic and parasympathetic nervous system, their balance and reaction to stimuli adapt the organ’s function (like blood pressure, heart rate, respiration, metabolism) to the actual needs. Prolonged or extreme stimuli may upset this balance, and result in so called acute autonomic dysfunction causing functional and sometimes morphological organ abnormalities, which can rarely lead to fatal consequences. Such manifestations in the heart can be various arrhythmias, myocardial infarction, takotsubo cardiomyopathy and sudden death [33].

Sleep deprivation is accompanied by a rise in sympathetic tone to maintain arousal and counteract parasympathetic impulses driving the body to a relaxing, regenerative state [34], ultimately resulting in a mentioned imbalance and autonomic disfunction. It can also be a part or trigger of acute or chronic stress reaction.

Gaming itself produce stress, according to cardiologist Robert S. Eliot, (M.D. at the University of Nebraska Medical Center), who used a video game (Pong) to replicate stressful situations in more than 1000 patients. Heart rate increases of 60 beats per minute and blood pressures as high as 220 was often observed within one minute of starting a computer game, without the patients being aware of that [32]. Similarly, increase in heart rate and blood pressure was observed in eSport athletes, as well [35, 36], although such high alteration was not pronounced. Acute elevation of blood pressure can trigger lethal arrhythmia in some cases [37]. Presence of stress during eSport activity can be demonstrated by the elevation of stress hormones [38], just like during physical sports [39].

Acute emotional distress (especially anger-like stress) may provoke ventricular arrhythmia, as well as acute myocardial infarction [40, 41], but stress due to assault [42] or natural disasters are reported as triggers for SCD, as well [43]. 20 to 40% of sudden cardiac deaths may be precipitated by acute emotional stressors. A gamer may feel strong anxiety in an unfamiliar situation, panic in a high risk-high stake scenario, frustration when failing a challenge and losing progression or rage when repeatedly losing or getting “owned”.

Even if the game is not especially stressful, extremely long gaming session could be compared to overtime at working, leading to exhaustion. In Japan several hundred cases were reported where people have died while repeatedly working overtime (termed as karoshi: death by overwork) [44, 45].

Dr. Su mentioned air pollution as another possible factor: Taiwan’s air relative humidity usually remains at 60–90%, that help fungi, bacteria and dust mites to flourish in a confined space. Taiwanese internet cafés were typically crowded, smoking regulation was more liberal at that time, poor ventilation and air conditioning may cooled but not improved the quality of air [32]. Air-pollution index in internet cafés often exceeded safe levels. Severe air pollution was shown to trigger coagulation and thrombosis, increase heart rate and reduce heart rate variability, cause endothelial dysfunction, arterial vasoconstriction, apoptosis, and hypertension. In chronic exposures these contribute to the progression of atherosclerosis, but even acute exposures can lead to autonomic nervous system imbalance, plaque instability, and trigger acute cardiovascular events (myocardial ischemia and infarction, stroke, heart failure, arrhythmias, and sudden death) [46]. Air pollution may contribute to acute autonomic dysfunction, as well, just like sleep deprivation, acute and chronic, emotional or physical stress, which may explain the sudden unexpected/unexplained death of gamers.

## Limitations

It is unsure, how complete the search for cases was. A death case is more likely to be reported if it happens in public, happens to a celebrity or the case has extreme characteristics. In several cases, relatives provided information about death cases that happened at home, especially if they wanted to message to the community. It is quite possible though, that others wanted to mourn in private and refused media presence. A source mentioned “In 2006, ten South Koreans reportedly died from blood clots suffered while sitting for extended periods playing games in a local PC Bang” [S5], though we had no information about these nameless cases either. We could only recover information from English language sources, like international electronic newspapers. If a case was not interesting enough for international media, and appeared only in local language news, it could have been lost to us. The demonstrated cases were reported in many news portals and added to various compilations. These “overhyped” cases appear in the frontline of an internet search, and they are the realistically visible ones. Others with less clicks, are squeezed out from the first few thousand search results, and are lost from sight among the several hundred million finds.

Quality of source data was sometimes debatable. Information from different sources were highly redundant with hardly any novelty. Sources must have shared or taken information from each other or worked with the same limited communiques. Details were often poor, the exact cause of death was unconfirmed or speculative, autopsy reports were rarely available.

## Conclusion

We aimed to review death cases linked to the action of playing video games. In several cases extreme length of sedentary gaming led to deep vein thrombosis and fatal pulmonary embolism. Lethal arrythmia due to acute autonomic dysfunction brought on by stress and sleep deprivation seem to be a frequent cause, as well. It mostly involved adolescent males, who were playing action-rich games. Most of them probably had internet gaming disorder. Incidence of gaming related death itself seems to be relatively low, much lower than sport related death in its risk population, but we should be aware that besides the low direct risk to life, gaming addiction can have adverse criminal and economical consequences, as well, and awareness of the problem should be maintained and prevention should be enforced.

## Supplementary Information

**Supplementary material 1.**

## Data Availability

Not applicable. No relevant numerical data was used. Most relevant sources for cases are linked in the Supplementary material.
