# Supplementary material for: Sudden gamer death: non-violent death cases linked to playing video games
Source: BMC Psychiatry. 2022 Dec 23;22:824. doi: 10.1186/s12888-022-04373-5 (PMC9789564; doi:10.1186/s12888-022-04373-5)
Supplement: Supplementary file 1 — Supplementary material 1. [file 12888_2022_4373_MOESM1_ESM.docx]

**Supplementary material**

**Brief description of death cases:**

Case No. 0-1.

Multiple sources mention **Jeff Dailey** (19-year-old male) and **Peter Burkowski** (18-year-old male) as the first video game-associated deaths *in 1981 and 1982* respectively, both guys having died playing “Berzerk” in an arcade in Chicago. Details about Burkowski’s death are more available: he was a good student, free of drinking or drugs, apparently healthy. He set up two top-ten scores in the game within 15 minutes, then turned to another arcade machine, collapsed, and died – 30 minutes within arriving to the arcade. Autopsy reported scar tissue on his heart, and stress of playing might have had an impact. Details about Dailey are much more lacking and controversial, his case might even be an urban legend [S1,S2,S3].

Case No. 2.

Some sources report **Kim Kyung-Jae** (24-year-old male) the first human to die from playing a video game too much. He died of deep vein thrombosis and pulmonary embolism in *October 2002* in an internet café in Gwangju (South Korea), after playing the video game “MU” for 86 hours with pauses only to purchase cigarettes and to use the bathroom [S4,S5,S6].

Case No. 3.

28-year-old male, **Seung Seop Lee** died on *5 August 2005* in an internet café in Taegu (South Korea). He was reported to be a gaming addict (often playing 14-18 hours a day), and recently lost job and broke up with his girlfriend. Lee was playing StarCraft for 50 hours with brief naps, reportedly ate and drank very little (if at all) and left his PC only to take restroom breaks. He fell off his chair onto the floor; a witness recalls he was conscious with closed eyes. He was rushed to nearby hospital where he died a few hours later presumably due to heart failure brought on by exhaustion and dehydration [S7,S8,S9].

Case No. 4.

26-year-old male from Jinzhou (Northern China) named **Zhang**, addicted to video games, died on *24 February 2007*. He had been playing World of Warcraft at home for seven days during the lunar new year holiday, stopping only for eating, toilet breaks, and a few hours of sleep in his bed. Parents witnessed him twitching and slumping over his computer screen. Resuscitation attempt was unsuccessful. The death was supposed to be a consequence of heart failure due to his obesity (ca. 150 kg), poor blood circulation, and oxygen deprivation from sitting for too long [S10,S11].

Case No. 5.

30-year-old **Chinese man** dropped dead at a cybercafé in Guangzhou (China) on *15 September 2007* after he had been playing games online for 3 days without considerable breaks. Paramedics tried to revive him, unsuccessfully. Further information was not disclosed [S12,S13].

Case No. 6.

25-year-old „fit and healthy” male, **Tim Eves** collapsed and died while 'jogging' on a Nintendo Wii Fit games console on *4 March 2009* in Hopton-on-Sea (UK). He had just returned from Portugal a few hours before. Sudden Arrhythmia Death Syndrome was considered as cause of death [S14,S15].

Case No. 7.

In February 2011 a 30-year-old **Chinese man** died at an internet cafe on the outskirts of Beijing after a three-day online gaming session without eating or sleeping [S16,S17].

Case No. 8.

20-year old male, **Chris Staniforth** from Sheffield (UK) often played “Halo” online on his Xbox for periods up to 12 hours. He died in *May 2011* after an interview at a JobCentre (he picked up a packet of chewing gum from the ground, he jolted back and began to spasm). Previously he was woken in the night by a ‘strange feeling’ in his chest. Cause of death was deep vein thrombosis and pulmonary embolism. Immobility was presumed to be a triggering factor [S18,S19].

Case No. 9.

On 22 July 2011, 13-year-old girl, **Anna-Lee Kehoe** from Bridport (UK), was playing on her Xbox, when she stood up, complained about shortness of breath, and collapsed. 10 minutes before, she had gone to the toilet, and had been fine. She was resuscitated but remained brain-dead. The girl was known to have asthma, but otherwise was perfectly healthy. Heart attack was considered as cause of death [S20,S21,S22].

Case No. 10.

23-year-old male, **Chen Rong-Yu**, died in an internet cafe on *1 February 2012* in New Taipei City (Taiwan). He spent 23 hours almost continuously playing “League of Legends”, stopping occasionally only to rest his head on the table in front of his monitor and sleep for a little while, then picked up his game where he had left off. Nine hours after one such episode an employee tried to wake him, but found him dead, still reaching for the keyboard.

Initial reports indicated he may have died due to cardiac arrest brought on by cold temperature, exhaustion, and lack of movement. According to his family he had been treated for a heart condition last year [S17,S23,S24].

Case No. 11.

18-year-old male, **Chuang** died on *15 July 2012* in Taiwan. He had been playing 40 hours with “Diablo III” in a private room of an internet cafe without eating or sleeping. He was found resting on the table. After the staff woke him up, he took a few steps and collapsed, then died shortly after arrival to hospital. Thromboembolism due to spending such a long time seated was suspected as cause of death [S16,S25,S26].

Case No. 12.

In August 2013, 35-year-old man, **Chang** was found dead in his seat after playing games for 10 hours at an internet cafe in Hualien City (Taiwan) [S27].

Case No. 13.

In February 2014, 40-year-old man, **Wang**, died after playing games for 13 hours at an internet cafe in Greater Kaohsiung (Taiwan). Attendants found Wang dead, but his eyes were still open and staring at the screen as though he were still playing [S27].

Case No. 14.

38-year-old man, **Chu**, was found dead in the bathroom of a New Taipei (Taiwan) internet cafe on *1 January 2015*, after a five-day-straight marathon of computer gaming. Except for going to the bathroom, taking cigarette breaks and occasional quick meals, the man did not leave his seat. Medicines to treat liver disease and gall stones were found in Chu’s belongings. Police officers said that Chu likely perished because of his medical condition, which was exacerbated by fatigue and physical exhaustion after five days playing computer games [S27].

Case No. 15.

32-year-old male, **Hsieh** in Kaohsiung (Taiwan), unemployed for longer time, regularly spent entire days in an internet cafe gaming. He used to take naps sitting in the chair or laying on the desk there. On *8 January 2015* he was found unresponsive laying on the keyboard, probably dead for several hours, after having played 3 consecutive days with combat computer games. Security camera footage showed Hsieh struggled with chest pains before collapsing. Cause of death was considered cardiac arrest, cold temperature, lack of movements and over-exhaustion from playing for so many hours non-stop being possible contributing factors [S28,S29,S30,S31,S32].

Case No. 16.

In March 2015, 24-year old male, **Wu Tai** died in an internet café in Shanghai (China) after sitting and playing 19 hours without a break with World of Warcraft. He was sitting in front of a computer, then started to cough violently. Witness saw him pale and to cough up blood, then he slumped in his chair. Paramedics tried to resuscitate him, unsuccessfully [S33,S34].

Case No. 17.

17-year-old boy, **Rustam** in Russia's Republic of Bashkortostan died on *1 September 2015*. The boy had been playing with “Defence of the Ancients” for 2000 hours in the past year and a half. After breaking his leg, and staying at home, he played it for 22 days almost all the time, stopping only to take a nap and grab a snack. Cause of death was presumed thromboembolism due the broken leg and not moving around at all [S35,S36].

Case No. 18.

35-year-old male **Brian Vigneault** (alias Poshybrid) died on *19 February 2017* in Virginia (USA). He regularly made marathon gaming streams on Twitch. In this case he announced a 24-hour-long stream to raise money for charity. He was playing “World of Tanks”, repeatedly dozing off towards the end. At 22 hours he left for a cigarette break (he was known to be a heavy smoker) and never returned. Despite other speculations, cause of death later was reported as fentanyl overdosing [S37,S38,S39].

Case No. 19.

On 2018 October 29, 21-year-old male Swedish “Fortnite” pro-gamer and streamer **Bogdan Akh** passed away during sleep the night after finishing at 18th place at the Fall Skirmish finals at TwitchCon in San Jose (USA). His teammate found him dead in the morning. They have been playing Fortnite intensively every single day for the last two months to practice for TwitchCon. Cause of death was not revealed [S40,S41,S42].

Case No. 20.

11-year-old boy **Fahad Fayyaz** died on *5 February 2019* in Lahor (Pakistan) while playing “Fortnite”. Previously he returned home from school in a happy mood and had showed no signs of any sort of stress. Parents later found him unconscious in his room with a controller in his hand. He wanted to play with his friends, but one of them couldn’t join, which Fahad commented as “If I lose this game, I will get a heart attack.” He was reported to be addicted to this game, despite his parents trying to stop him, he would still play for hours. Heart attack was considered as cause of death [S43,S44].

Case No. 21.

**Natsupon Arunrat** (32-year-old male) died on *27 April 2019* in an internet cafe in Samut Prakan (central Thailand). He started gaming at 4 am and was found dead in the evening probably 4 hours after having died, laying back on the chair, his mouth covered with blood from biting his tongue. He regularly spent the whole day there. His mother reported that he had been suffering from heart disease and high blood pressure for many years and could not work, and he had been addicted to computer games. Cardiac arrest was proposed [S45].

Case No. 22.

17-year-old male, **Piyawat Harikun** in Udon Thani (northern Thailand) was found dead, collapsed to the floor from his computer chair. During his school holiday, the boy had had all-night gaming sessions with multiplayer combat games, which he continued through the day until being found dead on the afternoon of *4 November 2019*. Medics said the teenager died from a stroke, which they believe was caused by playing the computer constantly through the night [S46,S47,S48,S49].

Case No. 23.

In *October 2020*, 12-year-old boy **Muhammad** from Egypt was found unresponsive (and dead before he arrived to the hospital) after playing “PlayerUnknown’s Battlegrounds” for hours on his mobile phone. The boy was reported to be obese and addicted to the game. Coroner’s preliminary examination attributed the cause of death to cardiac arrest from a sudden increase in blood pressure [S50].

Case No. 24.

A 16-year-old private school boy, **Dharshan**, collapsed and died on *1 February 2021* in Puducherry (India), while playing “Fire Wall” on his mobile phone for about 4 hours after school. Resuscitation attempt was unsuccessful. Autopsy revealed cerebral haemorrhage. Stroke expert neurology professor presumed unlikely that indulgence in video games was the reason for death in this case, though a surge in adrenaline spiking heart rate and blood pressure due to playing games, just as due to overexertion of any kind can be fatal by an underlying vascular malformation [S51].

**Brief description of video games:**

World of Warcraft is a *role-playing game* (RPG) [S53], where player control an avatar completing quests, gathering and crafting items and fighting foes to become better, richer, stronger in the game. The massive world to explore and countless things to do binds the player into the game for endless hours. A big part of the game is about combat, where player must use his offensive skills/abilities/spells to defeat his opponents and use his defensives to keep himself and allies alive. Fighting can be intense and really challenging against boss monsters and other players, requiring all attention and mental focus for quickly responding to the opponent’s actions, optimally timing and using the skills, and precisely executing moves. *Action RPGs* like Mu and Diablo are heavily combat focused versions of RPGs. Defence of the Ancients and League of Legends are *multiplayer online battle arena* (MOBA) games [S54], where two groups of player-avatars with unique set of skills aim to destroy the opponents base while protecting their own. Besides the intensive and short clashes, player must recognize the opportunities, outsmart, outmaneuver, snipe and hinder the opponent, exploit the environment, find the weak spot and time window to strike, share up the duties to several fronts or gather up to overpower one location as necessary. Besides his combat skills, the player must think and plan rapidly and adapt to his group’s and the opponent’s strategy, without a moment to pause till the game is over.

Starcraft is a real time strategy (RTS) game [S55], where the player must create and improve a base, expand to build up a sufficient economy to fuel the production of military units, which he moves to destroy the opponent’s base. A good player should constantly scout the opponent to predict his movements, and set his arsenal for the best counter. He must lead attacks on multiple fronts to outmaneuver defenses and even sacrifice squads to cripple the opponent’s economy to get the upper hand, while trying to deny the opponent doing the same. In the battles micromanaging individual units is suggested for best efficiency. And doing all these parallel in real time, needs incredible multitasking, fast decision making and execution. In these fast paced games high tier players usually have an average action-per-minute (such as selecting units or issuing an order) value of 300-350, jumping even higher during intense battle sequences.

In several combat games player control his avatar seeing through its eyes (first person shooters (FPS) [S56], like Halo, Firewall) or from behind it (third person shooters (TPS) [S57], like PlayerUnknown’s Battlegrounds, Fortnite). His aim is to kill the opponent player-avatars, or need to do this to reach his main goal (capture/defend the base, capture the flag etc.). Besides lightning fast reflexes and precise aim, the player must constantly stay focused to spot opponents in his limited field of view, know and exploit the terrain to ambush, escape and maneuver.

**References for Supplementary material:**

1. Kiesling S. Death of a video gamer. Video Games. 1982. p. 14-15. <http://home.hiwaay.net/~lkseitz/cvg/death.html>. Accessed 11 May 2022.
2. VGChartz Staff. This week in gaming hystory (Jan 17-23). News. 2010. <https://www.vgchartz.com/article/6598/this-week-in-gaming-history-jan-17-23/>. Accessed 11 May 2022.
3. Dauber. Any more details about Jeff Dailey's Berzerk death? AtariAge. 2012. <https://atariage.com/forums/topic/207011-any-more-details-about-jeff-daileys-berzerk-death/>. Accessed 11 May 2022.
4. Byung-hee L. Computer dies, world's first case. SBS News. 2004. <https://news.sbs.co.kr/news/endPage.do?news_id=N0311617389>. Accessed 11 May 2022.
5. Watkins SC. The Young and the Digital. Chapter 6: Hooked. Rethinking the internet addiction debate. Beacon Press. 2010. <https://books.google.hu/books?id=dhXhUs4Zh08C&pg=PT131&lpg=PT131&dq=Kim+Kyung-Jae+died+2002&source=bl&ots=o0mgIgYtnB&sig=ACfU3U01WQZEPElQTJJ9Izas1iHE7popMg&hl=hu&sa=X&ved=2ahUKEwj2utWAsfP0AhUN26QKHXpqAr8Q6AF6BAgWEAM#v=onepage&q=Kim%20Kyung-Jae%20died%202002&f=false>. Accessed 11 May 2022.
6. Gluck C. South Korea's gaming addicts. BBC news. 2002. <http://news.bbc.co.uk/2/hi/asia-pacific/2499957.stm>. Accessed 11 May 2022.
7. Naughton P. Korean drops dead after 50 hour gaming marathon. The Times. 2005. <https://www.thetimes.co.uk/article/korean-drops-dead-after-50-hour-gaming-marathon-skd6c0w7hbm>. Accessed 11 May 2022.
8. S Korean dies after games session. BBC News. 2005. <http://news.bbc.co.uk/2/hi/technology/4137782.stm>. Accessed 11 May 2022.
9. Xallium. Lee Seung Seop. Starcraft Wiki. 2007. [https://starcraft.fandom.com/wiki/Lee Seung Seop](https://starcraft.fandom.com/wiki/Lee%20Seung%20Seop). Accessed 11 May 2022.
10. Spencer R. Man dies after 7-day computer game session. The Telegraph. 2007. <https://www.telegraph.co.uk/news/worldnews/1544131/Man-dies-after-7-day-computer-game-session.html>. Accessed 11 May 2022.
11. Yong W. Obese online gamer dies playing. China Daily. 2007. [http://www.chinadaily.com.cn/china/2007-02/28/content 815396.htm](http://www.chinadaily.com.cn/china/2007-02/28/content%20815396.htm). Accessed 11 May 2022.
12. Chinese Man Drops Dead After 3-Day Gaming Binge. Fox News. 2015. <https://www.foxnews.com/story/chinese-man-drops-dead-after-3-day-gaming-binge>. Accessed 11 May 2022.
13. Reuters Staff. Man in China dies after three-day Internet session. Internet News. 2007. <https://www.reuters.com/article/us-china-internet-death/man-in-china-dies-after-three-day-internet-session-idUST16999720070917>. Accessed 11 May 2022.
14. ’Fit and healthy’ 25-year old died using Nintendo Wii Fit game. Telegraph. 2009. <https://www.telegraph.co.uk/news/uknews/5074714/Fit-and-healthy-25-year-old-died-using-Nintendo-Wii-Fit-game.html>. Accessed 11 May 2022.
15. Daily Mail Reporter. 'Healthy' man, 25, collapses and dies playing Wii Fit game. Daily Mail. 2009. <https://www.dailymail.co.uk/news/article-1165796/Healthy-man-25-collapses-dies-playing-Wii-Fit-game.html>. Accessed 11 May 2022.
16. Rudd A. Diablo death: Teenager dies after playing video game for 40 hours without eating or sleeping. Mirror. 2012. <https://www.mirror.co.uk/news/world-news/diablo-iii-death-teenager-dies-1147472>. Accessed 11 May 2022.
17. Parkin S. The sometimes fatal attraction of video games. The Guardian. 2015. <https://www.theguardian.com/technology/2015/aug/09/who-killed-the-video-gamers-simon-parkin-taiwan>. Accessed 11 May 2022.
18. Twomey R. Xbox addict, 20, killed by blood clot after 12-hour gaming sessions. Daily Mail. 2016. <https://www.dailymail.co.uk/news/article-2020462/Xbox-addict-20-killed-blood-clot-12-hour-gaming-sessions.html>. Accessed 11 May 2022.
19. Gamer Chris Staniforth's death blamed on DVT. BBC News. 2011. <https://www.bbc.com/news/uk-england-south-yorkshire-14350216>. Accessed 11 May 2022.
20. Coles J. Anna-Lee, 13, dies as she plays Xbox. The Sun. 2011. <https://www.thesun.co.uk/archives/news/686460/anna-lee-13-dies-as-she-plays-xbox/>. Accessed 11 May 2022.
21. Nguyen L. 15 People Who Have Died Playing Video Games. The Gamer. 2017. <https://www.thegamer.com/15-people-who-have-died-playing-video-games/>. Accessed 11 May 2022.
22. Daily Mail Reporter. 'Mum, I can't breathe': Tragic last words of 13-year-old girl who suffered heart attack as she played Xbox. Daily Mail. 2011. <https://www.dailymail.co.uk/news/article-2019370/Mum-I-breathe-Tragic-words-13-year-old-girl-suffered-heart-attack-played-XBox.html>. Accessed 11 May 2022.
23. Fahey M. Gamer Dies in Taipei Internet Cafe, Nine Hours Later Someone Notices. Kotaku. 2012. <https://kotaku.com/gamer-dies-in-taipei-internet-cafe-nine-hours-later-so-5881944>. Accessed 11 May 2022.
24. Cooper R. Gamer lies dead in internet cafe for 9 hours before anyone notices. Daily Mail. 2012. <https://www.dailymail.co.uk/news/article-2096128/Gamer-lies-dead-Taiwan-internet-cafe-9-HOURS-notices.html>. Accessed 11 May 2022.
25. Taiwan teen dies after gaming for 40 hours. News.com.au. 2012. <https://www.news.com.au/world/breaking-news/taiwan-teen-dies-after-gaming-for-40-hours/news-story/dd352da6751e7d9812c8278a138ef6f6>. Accessed 11 May 2022.
26. Neagle C. Teenager dies after 40-hour video game binge. Network World. 2012. <https://www.networkworld.com/article/2222807/teenager-dies-after-40-hour-video-game-binge.html>. Accessed 11 May 2022.
27. Pan J. Man dies after five-day gaming binge. Taipei Times. 2015. <http://www.taipeitimes.com/News/front/archives/2015/01/03/2003608347>. Accessed 11 May 2022.
28. Hunt K. Man dies in Taiwan after 3-day online gaming binge. CNN World. 2015. <https://edition.cnn.com/2015/01/19/world/taiwan-gamer-death/index.html>. Accessed 11 May 2022.
29. Feeney N. Man Found Dead in Taiwan After Multi-Day Video Game Binge. Time. 2015. Available from: <https://time.com/3673534/taiwan-video-game-death/>. Accessed 11 May 2022.
30. Ryall J. Online gamer dies after three-day binge. The Telegraph. 2015. <https://www.telegraph.co.uk/news/worldnews/asia/taiwan/11354383/Online-gamer-dies-after-three-day-binge.html>. Accessed 11 May 2022.
31. Holley P. Taiwanese man dies after three days of nonstop gaming. The Washington Post. 2015. Available from: <https://www.washingtonpost.com/news/morning-mix/wp/2015/01/18/taiwanese-man-dies-after-three-days-of-nonstop-gaming/>. Accessed 11 May 2022.
32. Pan J. Man dies following marathon Internet cafe gaming binge. Taipei Times. 2015. <http://www.taipeitimes.com/News/taiwan/archives/2015/01/17/2003609460>. Accessed 11 May 2022.
33. Roberts G. Tragedy as computer gamer dies after 19-hour session playing World of Warcraft. Mirror. 2015. <https://www.mirror.co.uk/news/world-news/tragedy-computer-gamer-dies-after-5263046>. Accessed 11 May 2022.
34. Crawley D. Man dies after 19-hour World of Warcraft session. VentureBeat. 2015. <https://venturebeat.com/2015/03/05/man-dies-after-19-hour-world-of-warcraft-session/>. Accessed 11 May 2022.
35. McCrum K. Tragic teen gamer dies after 'playing computer for 22 days in a row'. Mirror. 2015. <https://www.mirror.co.uk/news/world-news/tragic-teen-gamer-dies-after-6373887>. Accessed 11 May 2022.
36. Morrow M. Russian teenager dies after playing online computer game ‘Defence of the Ancients’ for 22 days in a row. News Corp Australia Network. 2015. <https://www.news.com.au/world/europe/russian-teenager-dies-after-playing-online-computer-game-defence-of-the-ancients-for-22-days-in-a-row/news-story/7f178341c80c5896a9c8c315b8e5c9b6>. Accessed 11 May 2022.
37. Devlin K. The mysterious death of a live-streaming gamer. BBC News. 2017. <https://www.bbc.com/news/blogs-trending-39232620>. Accessed 11 May 2022.
38. Guarino B. Prominent gamer died during live-streamed attempt to play ‘World of Tanks’ for 24 hours. The Washington Post. 2017. <https://www.washingtonpost.com/news/morning-mix/wp/2017/02/23/va-man-died-during-marathon-attempt-to-play-video-game-for-24-hours/>. Accessed 11 May 2022.
39. Crane K. Gaming and fentanyl: How one addiction may feed into the other. WYDaily. 2017. <https://wydaily.com/health/2017/05/28/gaming-and-fentanyl-how-one-addiction-may-feed-into-the-other-health/>. Accessed 11 May 2022.
40. Cruz E. Fortnite pro-gamer Bogdan Akh passed away after participating in the game’s Fall Skirmish. Blasting News US. 2018. <https://us.blastingnews.com/gaming/2018/11/fortnite-pro-gamer-bogdan-akh-passed-away-after-participating-in-the-games-fall-skirmish-002766595.html>. Accessed 11 May 2022.
41. James, F. Bogdanakh, a professional Fortnite player competing in the Fall Skirmish at TwitchCon last month, passed away in his sleep during the tournament. Twin Galaxies. 2018. <https://www.twingalaxies.com/feed_details.php/3648/pro-fortnite-player-bogdanakh-passed-away-during-twitchcon-fall-skirmish>. Accessed 11 May 2022.
42. Vanni. Fortnite: Professional Bogdan dies at age 21. Game Guides. 2018. <https://games-guides.com/fortnite-professional-bogdan-dies-at-age-21/>. Accessed 11 May 2022.
43. Craig N. 11 Year Old Pakistani Kid Dies while playing Fortnite. Research Snipers News. 2019. <https://www.researchsnipers.com/11-year-old-pakistani-kid-dies-while-playing-fortnite/>. Accessed 11 May 2022.
44. Pakistani 11 Year Old Kid Died Of Heart Attack While Playing Fortnite. The Islamic Information. 2019. <https://theislamicinformation.com/news/pakistani-kid-died-heart-attack-fortnite/>. Accessed 11 May 2022.
45. Aldersley M. Gamer drops dead from a heart attack at his keyboard in Thai internet cafe - but nobody notices for four hours. Mail Online. 2019. <https://www.dailymail.co.uk/news/article-6972673/Gamer-dies-heart-attack-keyboard-Thai-internet-cafe-notices-four-hours.html>. Accessed 11 May 2022.
46. Fahey R. Video game addict, 17, is found slumped dead on his computer after suffering a stroke as he played at night in Thailand. Daily Mail. 2019. <https://www.dailymail.co.uk/news/article-7650671/Video-game-addict-17-slumped-dead-computer-Thailand.html>. Accessed 11 May 2022.
47. Piriyapol A. Teenage gaming addict dies from stroke 'caused by all-night sessions' at computer. Mirror. 2019. <https://www.mirror.co.uk/news/world-news/teenager-dies-suddenly-stroke-caused-20813891>. Accessed 11 May 2022.
48. Lockett J. KILLER ADDICTION. Gaming addict, 17, collapses and dies after frenzied all-night session as devastated dad finds his body next morning. The Sun. 2019. <https://www.thesun.co.uk/news/10282260/gaming-addict-dies-all-night-session-thailand/>. Accessed 11 May 2022.
49. Miller JR. Teen video game addict dies after marathon session: report. New York Post. 2019. <https://nypost.com/2019/11/05/teen-video-game-addict-dies-after-marathon-session-report/>. Accessed 11 May 2022.
50. Ibrahim M. KILLER ADDICTION. Egypt issues fatwa banning video game PUBG after boy, 12, dies of heart attack during hours-long session. The Sun. 2020. <https://www.the-sun.com/news/1567023/egypt-fatwa-pubg-video-game-boy-dies/>. Accessed 11 May 2022.
51. Puducherry teen collapses after playing video game for hours, brought dead to hospital. The Hindu. 2021. <https://www.thehindu.com/news/cities/puducherry/puducherry-teen-collapses-after-playing-video-game-for-hours-brought-dead-to-hospital/article33738661.ece>. Accessed 11 May 2022.
52. Current World Population. Worldometer. 2022. <https://www.worldometers.info/world-population/>. Accessed 11 May 2022.
53. Role-playing video game. Wikipedia. <https://en.wikipedia.org/wiki/Role-playing_video_game>. Accessed 11 May 2022.
54. Multiplayer online battle arena. Wikipedia. <https://en.wikipedia.org/wiki/Multiplayer_online_battle_arena>. Accessed 11 May 2022.
55. Real-time strategy. Wikipedia. <https://en.wikipedia.org/wiki/Real-time_strategy>. Accessed 11 May 2022.
56. First-person shooter. Wikipedia. <https://en.wikipedia.org/wiki/First-person_shooter>. Accessed 11 May 2022.
57. Third-person shooter. Wikipedia. <https://en.wikipedia.org/wiki/Third-person_shooter>. Accessed 11 May 2022.
58. Sandbox game. Wikipedia. <https://en.wikipedia.org/wiki/Sandbox_game>. Accessed 11 May 2022.
59. Adventure game. Wikipedia. <https://en.wikipedia.org/wiki/Adventure_game>. Accessed 11 May 2022.
